# Multiscale Quantum Harmonic Oscillator Algorithm for Multimodal Optimization

**DOI:** 10.1155/2018/8430175

**Published:** 2018-05-13

**Authors:** Peng Wang, Kun Cheng, Yan Huang, Bo Li, Xinggui Ye, Xiuhong Chen

**Affiliations:** ^1^School of Computer Science and Technology, Southwest University for Nationality, Chengdu, China; ^2^Chengdu Institution of Computer Application, China Academy of Science, Chengdu, China; ^3^University of Chinese Academy of Sciences, Beijing, China; ^4^School of Computer Science and Technology, Huaiyin Normal University, Huaian, China; ^5^School of Computer Science and Engineering, University of Electronic Science and Technology of China, Chengdu, China; ^6^Jiangsu Key Laboratory of Media Design and Software Technology, Jiangnan University, Wuxi, China

## Abstract

This paper presents a variant of multiscale quantum harmonic oscillator algorithm for multimodal optimization named MQHOA-MMO. MQHOA-MMO has only two main iterative processes: quantum harmonic oscillator process and multiscale process. In the two iterations, MQHOA-MMO only does one thing: sampling according to the wave function at different scales. A set of benchmark test functions including some challenging functions are used to test the performance of MQHOA-MMO. Experimental results demonstrate good performance of MQHOA-MMO in solving multimodal function optimization problems. For the 12 test functions, all of the global peaks can be found without being trapped in a local optimum, and MQHOA-MMO converges within 10 iterations.

## 1. Introduction

Many real-world optimization problems are multimodal optimization problems, such as classification problems in machine learning [1] and inversion of teleseismic waves [2]. Multimodal optimization problems always contain several high quality global or local solutions which have to be identified and the most appropriate solution should be chosen. Global optimization of a continuous multimodal function aims at finding its several global minima or the most appropriate solution, without being trapped in a local optimum. When facing complex multimodal optimization problems, traditional optimization methods, such as gradient descent, quasi-Newton method, and Nelder–Mead's simplex methods, which may exploit all local information in an effective way, can easily be trapped into the local optimum. If a point-by-point classical optimization approach is used for this task, the approach must have to be applied several times, each time hoping to find a different optimal solution. There are two main reasons for us to find such optima as many as possible. Firstly, an optimal solution currently favorable in the future may not remain to be so. With the knowledge of another optimal solution for the problem, users can simply switch to this new optimal solution when such a predicament occurs. Secondly, the sheer knowledge of multiple optimal solutions in the search space may provide useful insights to the properties of optimal solutions of the problem. Evolutionary algorithms (EAs) and particle swarm optimization (PSO) are used to tackle multimodal optimization problems.

Due to the population-based approach, EAs have natural advantage over classical optimization techniques. EAs maintain a population of candidate solutions, which are processed in every generation. If several distinct solutions can be preserved over all these generations, we will get multiple good solutions, rather than the only best solution. In recent years, there are several attempts to improve EAs so as to deal with multimodal fitness landscapes. Niching methods are widely used in genetic algorithms (GA), differential evolution (DE), and other evolutionary algorithms for multimodal optimization [1, 3–16].

Similar to EAs, PSO is also an iterative, population-based optimization technique. The principle of PSO is that each particle has learning ability. It can learn from itself (pbest) and its best neighbor (gbest). According to the learning approaches of particles, PSO can be divided into two models. One is the global model, the other one is the local model. In the local PSO model, each particle learns from the best particle in its neighborhood while in the global model every particle learns from the best particle in the whole population. To ensure different particles in the population converge into different optima in the solution space, the way of choosing neighborhood topology structure is crucial. This property leads to the application of PSO for multimodal optimization problems in recent years [17, 18]. Owing to PSO's features of easy-to-implement and robust adaptability, the PSO converges quickly. But once it gets stuck into the local optimum, it will be very difficult to get out from the local optimum. To overcome this problem, quantum theories are introduced into PSO system. Quantum behaved Particle Swarm Optimization (QPSO) is the quantum model of a PSO. In QPSO, individual particles have quantum behavior [19, 20]. Instead of position and velocity, wavefunction *ψ*(*x*, *t*) [21, 22] is used to depict the state of a particle in QPSO [23]. Though QPSO performs better in global optimization than standard PSO, it also has the problem of premature convergence.

A novel optimization algorithm named multiscale quantum harmonic oscillator algorithm (MQHOA) is proposed in 2013 [24]. The population parameter and sampling parameter are researched in [24]. The uncertainty principle, zero energy and quantum tunnel effect of MQHOA are researched in [25]. MQHOA was inspired by the wavefunction of quantum harmonic oscillator. It tranforms the optimization problems to find the low energy state of potential *V*(*x*) = *f*(*x*). The complex objective function's second order Taylor approximation is Harmonic oscillator potential. According to quantum theory, the wavefunction of quantum harmonic oscillator represents the distribution of optimal solution. Different spring coefficients in quantum harmonic oscillator correspond with different search scales. Different spring coefficients vary inversely with search scales.

MQHOA's structure is elegant and pithy. It only includes two iteration processes: Quantum harmonic oscillator process (QHO process) and multiscale process (M process). The goal of optimization problem is searching the lowest energy position *f*(*x*_best_) (where *x*_best_ is global minimum position). QHO process simulates the quantum harmonic oscillator annealing from high energy level to ground state. In M process, MQHOA chooses *σ* decreasing with a series of 1/2 to get an increased series spring coefficient. With the same *σ*, in QHO process, MQHOA defines a new wavefunction to get sufficient sampling points in the global optimal area. The new wavefunction is defined as the summation of Gaussian probability-density functions. MQHOA's wavefunction in scale *σ*_*s*_ is the sum of *k* Gaussian probability-density functions which take *k*_*i*_ as centers. It depicts the probability distribution of optimal solutions in domain. The equation can be written as

(1) $$\begin{array}{c} \psi^{s}\left(\;x\;\right)=\overset{k}{\underset{i=1}{\sum }}\frac{1}{\sqrt{2\pi }\sigma_{s}}e^{-{\left(x-k_{i}\right)}^{2}/2\sigma_{s}^{2}}. \end{array}$$

The experimental results of 15 typical two-dimensional test functions show that MQHOA performs well in finding global optima [24].

In this paper, we present a variant of MQHOA for multimodal optimization named MQHOA-MMO. Similar to PSO's local version, in the proposed MQHOA-MMO, for each scale, every sampling point just needs to compare with the sampling points which are of the same Gaussian distribution.

This paper is organized as follows.  Section 2 describe the framework of MQHOA-MMO. Test functions and comparasion algorithms are presented in Section 3. The results of experiments are discussed in Section 4. Finally, Section 5 concludes the paper.

## 2. The Framework of MQHOA-MMO

This section presents the framework of MQHOA-MMO. We define the symbols as follows:

- *k* is the number of swarms and Gaussian distributions.
- *m* is the number of sampling points of each Gaussian distribution.
- *σ*_min_ is the accuracy of optimization.
- *σ* is the standard deviation for all *x*_*i*_.
- *σ*_*k*_ is the standard deviation for all new *x*_*i*_.
- Δ*σ* is the absolute value of the difference between *σ* and *σ*_*k*_.
- *σ*_*s*_ is the current scale for iteration, the initial value is defined as the domain length.
- *S* = *x*_1_,…, *x*_*i*_,…, *x*_*k*_ is the swarm of *k* particles such that *x*_*i*_ indicates particle *i*  (*i* = 1,2, 3,…, *k*). *x*_*i*_ is randomly generated in domain. For every *x*_*i*_, generate *m*  *x*_*i*_^*q*^  (*q* = 1,2, 3,…, *m*), which are following probability distribution *N*(*x*_*i*_, *σ*_*s*_^2^). *k* × *m* sampling positions are needed by every iteration. *k* optimal positions are stored in *x*_*i*_.
- *x*_*i*_^best^ is the optimal position selected from *m* sampling positions *x*_*i*_^*q*^.
- *x*_best_′ is the optimal position the algorithm has found.

MQHOA-MMO includes just two nested iteration processes: QHO process and M process. In MQHOA-MMO, the QHO process is nested inside the M process. The convergence conditions of QHO process and M process are Δ*σ* < *σ*_*s*_ and *σ*_*s*_ ≤ *σ*_min_ respectively. The framework of MQHOA-MMO is described in Algorithm 1.

The elaborate interpretation of the framework of MQHOA-MMO is as follows:

1. Initialize *σ*_min_ = 0.00001, *d*_min_ = −10, *d*_max_ = 10. So *σ*_*s*_ = 20. Here, we choose *k* = 20, *m* = 200, the influence of the value *k* will be discussed in Section 4.1.
2. Randomly generate *x*_*i*_  (*i* = 1,…, 20) in [−10,10]. Calculate the standard deviation *σ* for all *x*_*i*_.
3. For each *x*_*i*_, generate *x*_*i*_^*q*^  (*q* = 1,…, 200), which are following probability distribution *N*(*x*_*i*_, *σ*_*s*_^2^).
4. Choose the optimal position *x*_*i*_^best^ from all *x*_*i*_^*q*^  (*q* = 1,…, 200) for each *x*_*i*_.
5. For each *x*_*i*_, *x*_*i*_ = *x*_*i*_^best^. Calculate the standard deviation *σ*_*k*_ for all new *x*_*i*_.
6. Calculate Δ*σ* = |*σ*_*k*_ − *σ*|.
7. Compare Δ*σ* and *σ*_*s*_. If Δ*σ* > *σ*_*s*_, return to step (3). If Δ*σ* < *σ*_*s*_, *σ*_*s*_ = *σ*_*s*_/2.
8. Compare *σ*_min_ and *σ*_*s*_. If *σ*_*s*_ > *σ*_min_, return to step (3). If *σ*_*s*_ < *σ*_min_, return *x*_best_′, *f*_best_.

According to the framework above, only two parameters (*k* and *m*) need to be set. The selection of *k* is discussed in Section 4.1.

In framework the superposition of *k* Gauss sampling areas constructs the wavefunction. Wavefunction written as equation (1) depicts the probability distribution of optimal solutions in domain. The changes of wavefunction in iterations are showed in Figure 5. In order to reduce the energy of system, *k* optimal positions *x*_*i*_^best^ are retained from *k* × *m* sampling positions. In QHO process, *σ*_*s*_ = *σ*_*s*_/2 transforms the system from high energy state at scale *σ*_*s*_ to ground state at scale *σ*_*s*_/2.

For high dimensional test functions, MQHOA-MMO can use two-dimensional array *x*_*ij*_ to store the *k* high dimensional central positions of Gauss sampling area (*N*(*x*_*ij*_, *σ*_*s*_)). Where *i* is dimension, *j* is the number of Gauss sampling areas. For every dimension, MQHOA-MMO calculates the value of *σ*_*k*_ and Δ*σ*. The QHO process at scale *σ*_*s*_ will end until Δ*σ* ≤ *σ*_*s*_ in every dimension.

## 3. Experimental Setup

In this section, we give a brief description of benchmark functions and comparison algorithms. Experimental setup is present at the end of this section.

### 3.1. Test Functions

The benchmark functions we choose are widely used in multimodal optimization. Some test functions have various characteristics, such as irregular landscape, symmetric or equal distribution of optima. The goals are thus to evaluate the ability to tackle a complicated problem, to validate its capacity to detect all the global peaks of a function. A brief description of the functions is listed in Table 1.

### 3.2. Comparasion Algorithms

To evaluate the performance of MQHAO-MMO, MQHAO-MMO is compared with the following standard multimodal evolutionary algorithms. MQHOA-MMO is marked as AL0.

  AL1 (IWO-*σ*-GSO [26]): the vasion weed optimization-*σ*-group search optimizers;
  AL2 (scma [27]): CAM-ES with Self-Adaptive Niche Radius;
  AL3 (cde [28]): the original crowding DE;
  AL4 (sde [29]): speciation-based DE;
  AL5 (ferpso [30]): fitness-Euclidean distance Ratio;
  AL6 (spso [31]): speciation-based PSO;
  AL7 (r2pso [31]): a lbest PSO with a ring topology, each member interacts with only its immediate member to its right;
  AL8 (r3pso [31]): a lbest PSO with a ring topology, each member interacts with only its immediate member to its left;
  AL9 (r2psolhc): the same as r2pso [32], but with no overlapping neighborhoods;
  AL10 (r3psolhc): the same as r3pso [32], but with no overlapping neighborhoods.

### 3.3. Experimental Environment and Criteria

MQHOA-MMO is coded in Matlab R2014 and the simulations are run on i5 CPU 2.9 GHz with 8 GB memory. Results are averaged over 30 independent runs. If the difference between a computed solution and a known global optimum is less than *ε*, the peak is considered to be found. The performance of all multimodal algorithms is measured in terms of the following two criteria:

1. Success rate: The percentage of runs in which an algorithm can detect all the global peaks.
2. Average peak number: Peak number found over 30 runs for each function.

## 4. Experimental Studies

The experimental studies and analyses are presented in this section. MQHOA-MMO had been run until *σ*_*s*_ < *σ*_min_ or the maximum number of function evaluation was exhausted.

### 4.1. Parameter Experiment

In this section, we examine the effectiveness and efficiency of MQHOA-MMO by applying it to selected four benchmark functions: Six-Hump Camel Back Function (E1-F4), Himmelblaus's Function (E1-F3), Hansen Function (E1-F8) and 2D Inverted Shubert Function (E1-F18). The numbers of solution individuals are 2, 4, 9, and 18, respectively.

**Figure 1:**
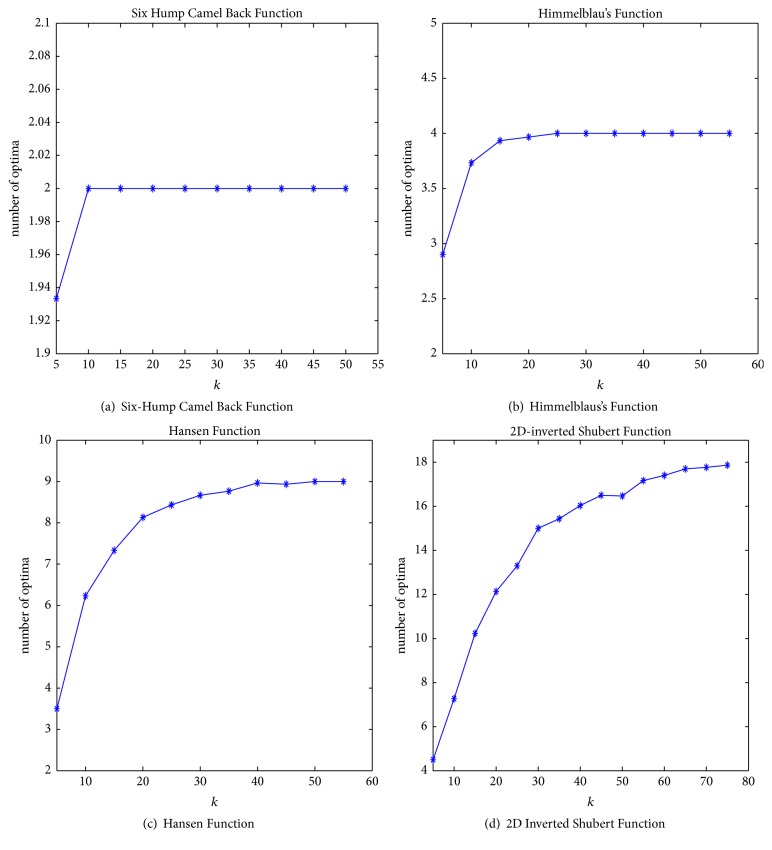
Relation of *k* and optimal solution with *m* = 200, *σ*_min_ = 10*e* − 6, and repeat time = 30.

In MQHOA-MMO, we generate initial population using two parameters: *k* and *m*. We choose different initial parameter *k* to test the impact on the ability of finding global optima. We run over 30 times for each *k* while *m* = 200.  Figure 1 contains four relation figures between the parameter *k* and the number of global optima which MQHOA-MMO can find. The initial *k* value begins at 5 and increases by 5 each time. According to Figures 1(c) and 1(d), while *k* is smaller than the number of global optima, MQHOA-MMO can only find part of global optima. With the increasing of *k*, we can find most global optima when the initial *k* is close to the number of global optima. When the number of optimal solutions is small, for example, less than 10, the increased *k* can find all the optimal solutions. But when the number of global optima is lager (more than 10), with the increase of *k*, the number of global optima which MQHOA-MMO can find also increases. When *k* increases to a certain number, the number of global optima which MQHOA-MMO can find is stable. When the domain is large, *k* should increase in a large scale more than ten times. We can make a conclusion that to find all the global optima the initial value of *k* should be larger than the number of the global optima. Besides, along with the increase of domain, the number of *k* should increase correspondingly.

### 4.2. Convergence Experiment

**Figure 2:**
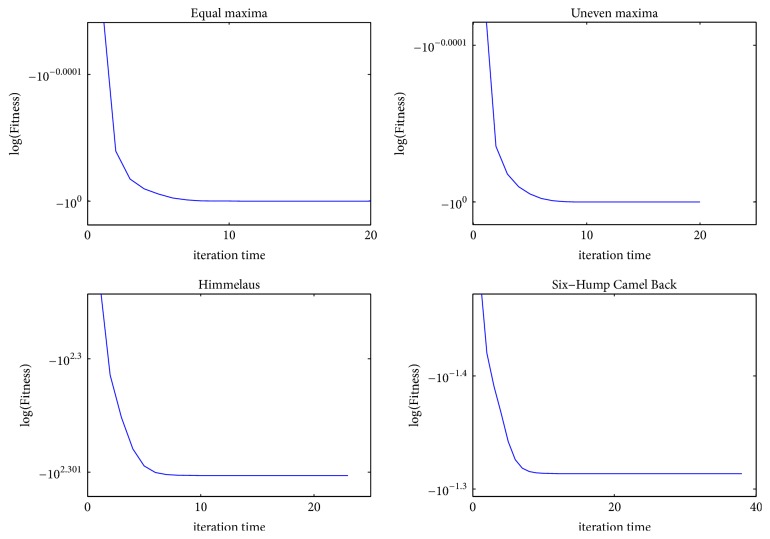
Convergence of *F*1–*F*4, where *k* = 50, *m* = 200, *σ*_min_ = 10*e* − 6, repeat time = 30.

**Figure 3:**
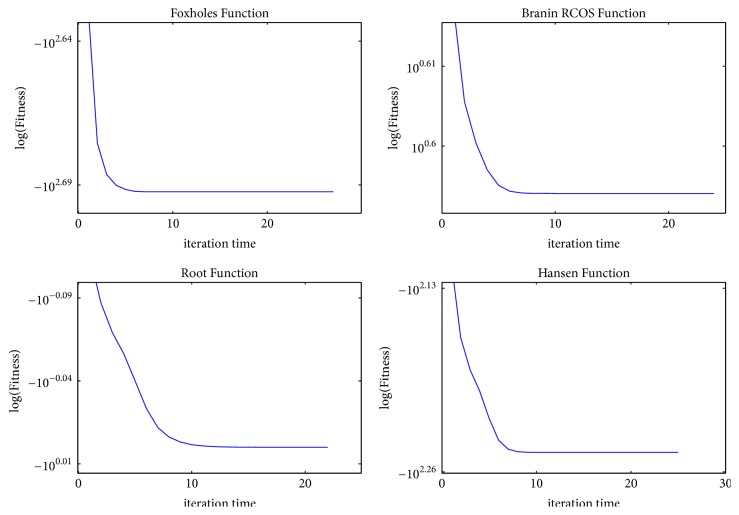
Convergence of *F*5–*F*8, where *k* = 50, *m* = 200, *σ*_min_ = 10*e* − 6, repeat time = 30.

**Figure 4:**
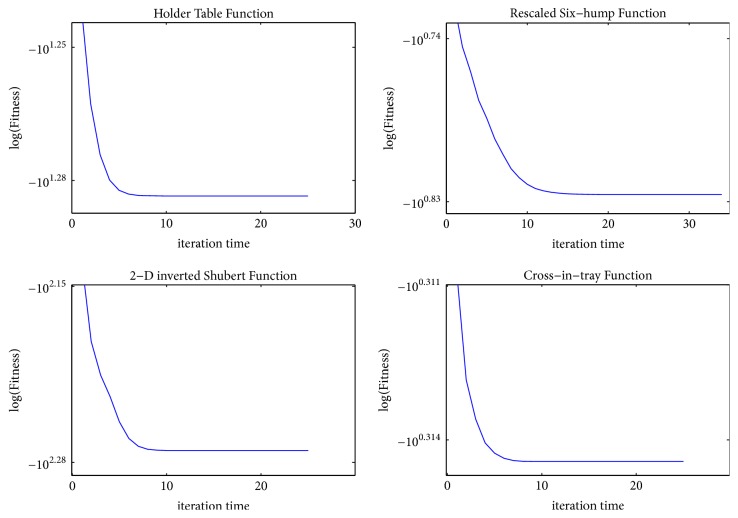
Convergence of *F*9–*F*11, where *k* = 50, *m* = 200, *σ*_min_ = 10*e* − 6, repeat time = 30.

To be more intuitive, we apply 12 benchmark test functions to demonstrate the convergence and verify the effectiveness of MQHOA-MMO. Figures 2, 3, and 4 show the relationship between fitness and iteration times. Fitness is the average of *k* optimum function values of test functions. The value of iteration times is the number of QHO processes. For the 12 benchmark test functions, we set parameters as follows: *k* = 50, *m* = 200, and *σ*_min_ = 10*e* − 6. Results are averaged over 30 independent runs.

From Figures 2–4, we find that almost all the functions have converged to several small areas before the tenth iteration. Some functions, such as *F*1, *F*2, *F*3, *F*5, *F*6, *F*9, and *F*12, can converge to several small areas at the fifth iteration.  Table 2 presents the iteration times of 12 test functions while *σ*_*s*_ < *σ*_min_ or the maximum number of function evaluations was exhausted. The results mean that MQHOA-MMO has a fast convergent ability, and for most test functions there is only one QHO process in each M process.

### 4.3. Changes of Wavefunction

In this section, we choose Hansen function (E1-F8) to present the changes of wavefunction in MQHOA-MMO. We set *k* = 35, *m* = 200, *d*_min_ = −10, and *d*_max_ = 10. The function is written as follows:

(2) Fx,y=∑i=15icos⁡i−1x−i∑j=15jcos⁡j+1y+j.

MQHOA-MMO's wavefunction is written as (1). To describe wavefunction clearly, we have defined three notions. First notion is incipient centers of *k* swarms, which are *x*_*i*_ used in QHO's first iteration for each *σ*_*s*_. Second notion is incipient wavefunction, which is the wavefunction of *x*_*i*_ used in QHO's first iteration for each *σ*_*s*_. The last notion is last wavefunction, which is the wavefunction of *x*_*i*_ used in QHO's last iteration for each *σ*_*s*_. After the last QHO iteration for each *σ*_*s*_, *σ*_*s*_ will be cut half to *σ*_*s*_/2.

**Figure 5:**
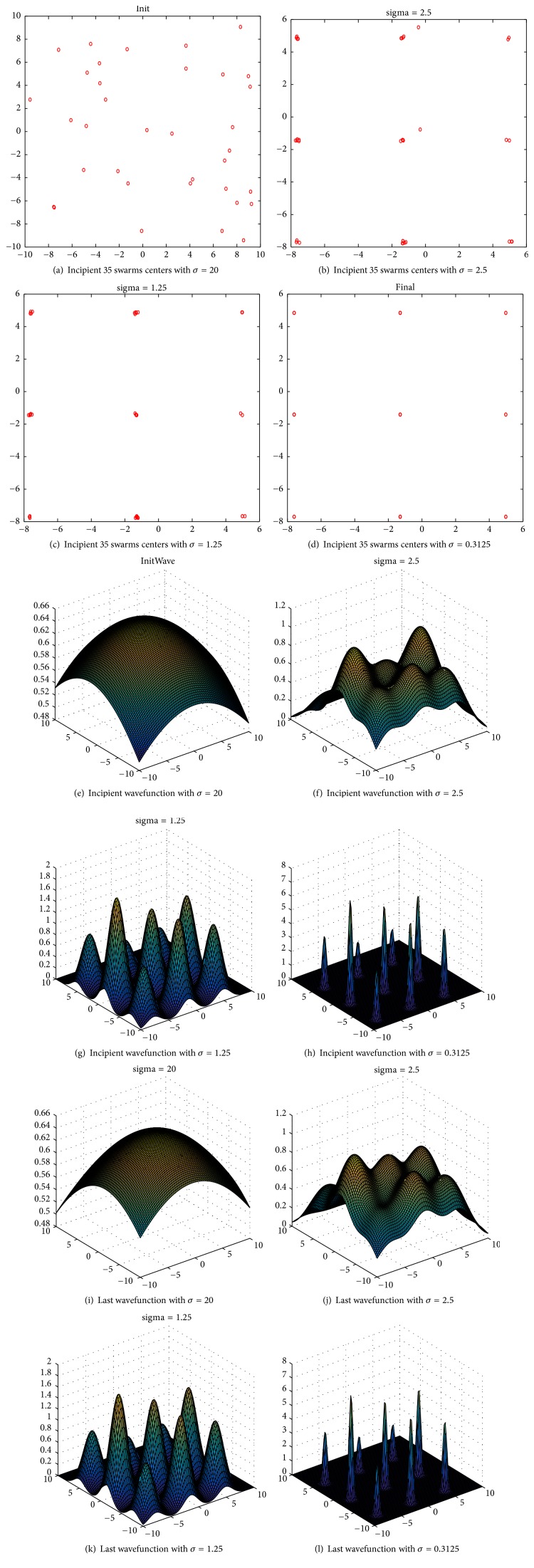
Changes of wavefunction in iterations.

Figure 5 presents the change of wavefunction in iterations. Figures 5(a)–5(d) show the incipient centers of *k* swarms with different *σ*_*s*_. Figures 5(e)–5(h) show the incipient wavefunctions with different *σ*_*s*_. Figures 5(i)–5(l) show the last wavefunctions with different *σ*_*s*_. With *σ*_*s*_ = 20, Figure 5(a) shows the incipient centers of swarms and Figure 5(e) shows the incipient wavefunction while the last wavefunction is shown in Figure 5(i). With *σ*_*s*_ = 2.5, Figure 5(b) shows the incipient centers of swarms, Figure 5(f) shows the incipient wavefunction, and the last wavefunction is shown in Figure 5(j). With *σ*_*s*_ = 1.25, Figure 5(c) shows the incipient centers of swarms, Figure 5(g) shows the incipient wavefunction, and the last wavefunction is shown in Figure 5(k). With *σ*_*s*_ = 0.3125, Figure 5(d) shows the incipient centers of swarms, Figure 5(h) shows the incipient wavefunction, and the last wavefunction is shown in Figure 5(l).

Figures of the same *σ*_*s*_ show the changes of wavefunctions in different QHO iterations. For example, Figures 5(g) and 5(k) show the changes in QHO iterations with *σ*_*s*_ = 1.25. As mentioned in Section 4.2 the number of QHO iterations is small in each M iteration, so the change of wavefunctions is not obvious in different QHO iterations with the same *σ*_*s*_. Figures of different *σ*_*s*_ give us the differences of wavefunctions in different M iterations. For example, Figures 5(i), 5(j), 5(k), and 5(l) show the changes of wavefunctions in M iterations. According to our definition, Figures 5(j) and 5(g) have the same centers of *k* swarms and the different *σ*_*s*_. From the wavefunctions, we also can find that if there is a large probability of the optimal solution at a certain point, the point will be more attracted in this area, according to Gauss sampling law. It means that the higher probability is that the optimal solution is in the region. With the decrease of *σ*_*s*_, the probability distribution of particles is more and more concentrated. This is the principle that MQHOA-MMO uses multiscale to implement the precision of the algorithm.

### 4.4. Comparison Experiments

In this section, we will present a detailed discussion on the performance of various algorithms that were chosen in the comparative study. We use 6 challenging functions of various characteristics to evaluate the MQHOA-MMO's performance. MQHOA-MMO runs until *σ*_*s*_ < *σ*_min_ or the maximum number of function evaluations was exhausted. The experimental results of other algorithms are quoted from [26]. All performances of MQHOA-MMO are calculated and averaged over 30 independent runs with *k* = 50, *m* = 200, and *σ*_min_ = 10*e* − 6. From Table 3, we can find that MQHOA-MMO's success rate can reach up to 100%. For some complex test functions, such as *F*3, *F*5, and *F*6, MQHOA-MMO can also get 100% success rate while some other algorithms even cannot find the global optima.

Table 4 shows the average number of global peaks detected by the MQHOA-MMO and other ten evolutionary multimodal optimization algorithms on test functions. Table 4 further indicates that MQHOA-MMO is able to detect the global optima in the test cases, and MQHOA-MMO can yield a good level of accuracy. IWO-*σ*-GSO is a new excellent combination algorithm. It can generate acceptable results over the test functions. SCMA can also generate good results over simple and low dimension. Their performance gradually becomes poor when the dimension increases. SDE algorithm shows very poor performance if the number of peaks has high accuracy. Other SDE could not be able to generate satisfactory solution. FERPSO is able to generate relatively satisfactory results in many test functions. For MQHOA-MMO, given suitable parameters, it can get all global optima.

## 5. Conclusion

In this paper, we proposed a multimodal optimization algorithm named MQHOA-MMO. We used wave function to locate the possibility positions of the optimal solutions. The experimental study undertook 12 distinct test functions with number of global peaks varying from 2 to 25. Through comparison of results which were obtained from optimization of several benchmark functions using MQHOA-MMO and other optimization algorithms with two criteria and results obtained from performance experiments it is revealed that MQHOA-MMO can detect all the global optimum in a fast effective, controllable, and higher accuracy. Furthermore, this algorithm can find the global optimum of multifunction without being trapped in local optimum. The experimental study clearly indicated that, in most of the test cases, performance of MQHOA-MMO remains statistically better than all the other algorithms compared with it. For some complex functions, MQHOA-MMO could not have a good success rate. Future research of MQHOA-MMO will be focused on the optimization of such complex functions and higher-dimensional functions.

## Figures and Tables

**Algorithm 1:**
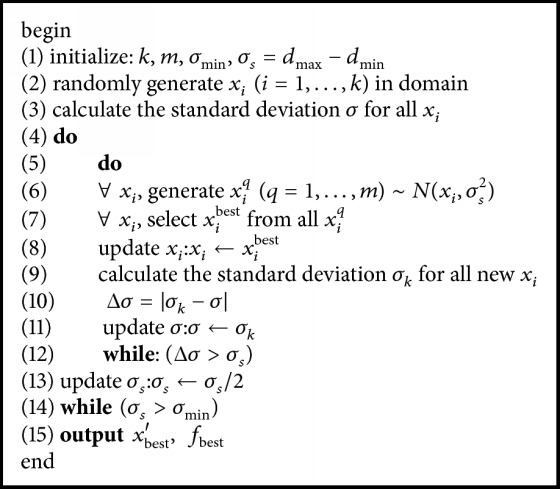
The framework of MQHOA-MMO.

**Table 1:** Benchmark function.

Function name	D	Range	Benchmark function	Optima
E1-F1: Equal maxima	1	*x* ∈ [0 1]	*F*1(*x*) = sin^6^⁡(5*πx*)	5
E1-F2: Uneven maxima	1	*x* ∈ [0 1]	*F*2(*x*) = sin^6^⁡(5*π*(*x*^3/4^ − 0.05))	5
E1-F3: Himmelblau's	2	*x* ∈ [−4 4]	*F*3(*x*, *y*) = 200 − (*x*^2^ + *y* − 11)^2^ − (*x* + *y*^2^ − 7)^2^	4
		*y* ∈ [−4 4]		
E1-F4: Six-Hump Camel	2	*x* ∈ [−1.9 1.9]	$F4\left(x,y\right)=\left(4-2.1x^{2}+\frac{x^{4}}{3}\right)x^{2}+xy+\left(-4+4y^{2}\right)y^{2}$	2
		*y* ∈ [−1.1 1.1]		
E1-F5: Shekel's foxholes	2	*x* ∈ [−65.54 65.54]	$F5\left(x,y\right)=500-\frac{1}{0.002+\sum_{i=0}^{24}\left(1/\left(1+i+{\left(x-a\left(i\right)\right)}^{6}+{\left(y-b\left(i\right)\right)}^{6}\right)\right)}$	1
		*y* ∈ [−65.54 65.54]		
E1-F6: Branin RCOS	2	*x* ∈ [−5 10]	$F6\left(x,y\right)={\left(y-\frac{5.1}{4\pi^{2}}x^{2}+\frac{5}{\pi }x-6\right)}^{2}+10\left(1-\frac{1}{8\pi }\right)\cos\left(x\right)+10$	3
		*y* ∈ [0 15]		
E1-F7: Root function	2	*x* = *x*_1_ + *ix*_2_	$F7\left(x\right)=\frac{1}{1+\left|x^{6}-1\right|}$	6
		*x* ∈ [−2 2]		
E1-F8: Hansen	2	*x* ∈ [−10 10]	*F*8(*x*, *y*) = ∑_*i*=1_^5^*i*cos⁡((*i* − 1)*x* − *i*) · ∑_*j*=1_^5^*j*cos⁡((*j* + 1)*y* + *j*)	9
		*y* ∈ [−10 10]		
E1-F9: Holder Table	2	*x* ∈ [−10 10]	$F9\left(x,y\right)=-\left|\sin\left(x_{1}\right)\cos\left(x_{2}\right)\cdot \exp\left(\left|1-\frac{\sqrt{x_{1}^{2}+x_{2}^{2}}}{\pi }\right|\right)\right|$	4
E1-F10: Rescaled Six-Hump Camel	2	*x* ∈ [−1.9 1.9]	$F10\left(x,y\right)=-\left(\left(4-2.1x^{2}+\frac{x^{4}}{3}\right)x^{2}+10xy+\left(-4+4{\left(10y\right)}^{4}\right)\right)$	2
		*x* ∈ [−1.1 1.1]		
E1-F11: 2D Inverted Shubert	2	*x*_1_ ∈ [−10 10]	$F11\left(\vec{X}\right)=-\prod_{i=1}^{D}\sum_{j=1}^{5}j\cos\left[\left(j+1\right)x_{i}+j\right]$	18
		*x*_2_ ∈ [−10 10]		
E1-F12: Cross-In-Tray	2	*X*_*i*_ ∈ [−10 10]	$F12\left(x\right)=-0.0001{\left(\left|\sin\left(x_{1}\right)\sin\left(x_{2}\right)\exp\left(\left|100-\frac{\sqrt{x_{1}^{2}+x_{2}^{2}}}{\pi }\right|\right)+1\right|\right)}^{0.1}$	4

**Table 2:** Iteration times (*N*) for 12 test functions, where *k* = 50, *m* = 200, *σ*_min_ = 10*e* − 6, *σ*_*s*_ = *d*_max_ − *d*_min_, and repeat time = 30.

*F*1	*F*2	*F*3	*F*4	*F*5	*F*6	*F*7	*F*8	*F*9	*F*10	*F*11	*F*12
20	20	20	20	27	24	22	25	25	22	25	25

**Table 3:** Success rates for test functions; the parameters for MQHOA-MMO are as follows: *k* = 50, *m* = 200, *σ*_min_ = 10*e* − 6, and repeat time = 30; the domain of definition is different with different functions.

Func	*ε*	AL0	AL1	AL2	AL3	AL4	AL5	AL6	AL7	AL8	AL9	AL10
*F*1	0.000001	100	100	92	28	72	84	88	92	88	100	92
*F*2	0.000001	100	100	88	28	60	100	92	88	72	92	92
*F*3	0.0005	100	88	72	0	72	72	0	24	28	24	24
*F*4	0.000001	100	60	60	0	100	96	0	60	56	52	60
*F*5	0.00001	100	100	88	52	32	100	56	88	76	72	60
*F*6	0.1	100	92	74	82	78	76	70	72	66	60	62

**Table 4:** Average number of peaks found for the test functions; the parameters for MQHOA-MMO are as follows: *k* = 50, *m* = 200, *σ*_min_ = 10*e* − 6, and repeat time = 30, and the domain of definition is different with different functions.

Func	*ε*	AL0	AL1	AL2	AL3	AL4	AL5	AL6	AL7	AL8	AL9	AL10
*F*1	0.000001	5	5	4.92	3.84	4.72	4.84	4.88	4.92	4.88	5	4.92
*F*2	0.000001	5	5	4.88	3.96	4.6	5	4.92	4.88	4.72	4.92	4.88
*F*3	0.0005	4	3.88	3.72	0.32	3.72	3.68	0.84	2.92	2.76	3	3.12
*F*4	0.000001	2	1.6	1.6	0.04	2	1.96	0.08	1.44	1.56	1.56	1.48
*F*5	0.00001	1	1	0.88	0.52	0.32	1	0.56	0.88	0.76	0.72	0.6
*F*6	0.1	3	2.88	2.56	2.76	2.72	2.64	2.48	2.52	2.44	2.36	2.40

## Data Availability

The data used to support the findings of this study are available from the corresponding author upon request.

## References

[1] Mahfoud S. W. (1995). Niching methods for genetic algorithms.

[2] Koper K. D., Wysession M. E., Wiens D. A. (1999). Multimodal function optimization with a niching genetic algorithm: A seismological example.

[3] Della Cioppa A., De Stefano C., Marcelli A. (2007). Where are the niches? Dynamic fitness sharing.

[4] Dasa S., Maity S., Qu B.-Y., Suganthan P. N. (2011). Real-parameter evolutionary multimodal optimization-A survey of the state-of-the-art.

[5] Sareni B., Krähenbühl L. (1998). Fitness sharing and niching methods revisited.

[6] Singh G., Deb K. Comparison of multi-modal optimization algorithms based on evolutionary algorithms.

[7] Jong K. A. D. (1975).

[8] Goldberg D. E., Richardson J. Genetic algorithms with sharing for multimodal function optimization.

[9] Harik G. R. (1995). Finding multimodal solutions using restricted tournament selection.

[10] Li J.-P., Balazs M. E., Parks G. T., Clarkson P. J. (2002). A species conserving genetic algorithm for multimodal function optimization.

[11] Mahfoud S. W. (1992). Crowding and preselection revisited.

[12] Beasley D., Bull D. R., Martin R. R. (1993). A Sequential Niche Technique for Multimodal Function Optimization.

[13] Bessaou M., Pétrowski A., Siarry P. (2000). Island Model Cooperating with Speciation for Multimodal Optimization.

[14] Parsopoulos K. E., Vrahatis M. N. (2004). On the computation of all global minimizers through particle swarm optimization.

[15] Yin X., Germay N. (1993). A fast genetic algorithm with sharing scheme using cluster analysis methods in multimodal function optimization.

[16] Petrowski A. Clearing procedure as a niching method for genetic algorithms.

[17] Juang Y.-T., Tung S.-L., Chiu H.-C. (2011). Adaptive fuzzy particle swarm optimization for global optimization of multimodal functions.

[18] Liu L., Yang S., Wang D. (2012). Force-imitated particle swarm optimization using the near-neighbor effect for locating multiple optima.

[19] Sun J., Feng B., Xu W. Particle swarm optimization with particles having quantum behavior.

[20] Sun J., Xu W., Feng B. Adaptive parameter control for quantum-behaved particle swarm optimization on individual level.

[21] Schweizer W. (2001).

[22] Levin F. S. (2002).

[23] Coelho L. D. S. (2008). A quantum particle swarm optimizer with chaotic mutation operator.

[24] Wang P., Huang Y., Ren C., Guo Y. M. (2013). Multi-scale quantum harmonic oscillator for high-dimensional function global optimization algorithm.

[25] Wang P., Huang Y. (2015). Physical model of multi-scale quantum harmonic oscillator optimization algorithm.

[26] Roy S., Islam S. M., Das S., Ghosh S. (2013). Multimodal optimization by artificial weed colonies enhanced with localized group search optimizers.

[27] Shir O. M., Emmerich M., Bäck T. (2010). Adaptive niche radii and niche shapes approaches for niching with the CMA-ES.

[28] Shen D., Li Y. (2013). Multimodal optimization using crowding differential evolution with spatially neighbors best search.

[29] Li X. Efficient differential evolution using speciation for multimodal function optimization.

[30] Seo J.-H., Im C.-H., Heo C.-G., Kim J.-K., Jung H.-K., Lee C.-G. (2006). Multimodal function optimization based on particle swarm optimization.

[31] Li X. (2004). Adaptively choosing neighbourhood bests using species in a particle swarm optimizer for multimodal function optimization.

[32] Li X. (2010). Niching without niching parameters: particle swarm optimization using a ring topology.

